# Assessing *in vivo* and *in vitro* biofilm development by *Streptococcus dysgalactiae* subsp. *dysgalactiae* using a murine model of catheter-associated biofilm and human keratinocyte cell

**DOI:** 10.3389/fcimb.2022.874694

**Published:** 2022-07-19

**Authors:** Cinthia Alves-Barroco, Ana Maria Nunes Botelho, Marco Antonio Américo, Sérgio Eduardo Longo Fracalanzza, António P. Alves de Matos, Márcia Aparecida Guimaraes, Bernadete Teixeira Ferreira-Carvalho, Agnes Marie Sá Figueiredo, Alexandra R. Fernandes

**Affiliations:** ^1^ UCIBIO - Applied Molecular Biosciences Unit, Dept. Ciências da Vida, NOVA School of Science and Technology, Caparica, Portugal; ^2^ i4HB, Associate Laboratory - Institute for Health and Bioeconomy, Faculdade de Ciências e Tecnologia, Universidade NOVA de Lisboa, Caparica, Portugal; ^3^ Instituto de Microbiologia Paulo de Góes, Universidade Federal do Rio de Janeiro, Rio de Janeiro, Brazil; ^4^ Centro de Investigação Interdisciplinar Egas Moniz (CiiEM), Egas Moniz - Cooperativa de Ensino Superior CRL, Quinta da Granja, Portugal

**Keywords:** host-pathogen interaction, bovine mastitis, bacterial cytotoxicity, biofilm development, SDSD pathogenesis

## Abstract

*Streptococcus dysgalactiae* subsp. *dysgalactiae* (SDSD) is an important agent of bovine mastitis. This infection causes an inflammatory reaction in udder tissue, being the most important disease-causing significant impact on the dairy industry. Therefore, it leads to an increase in dairy farming to meet commercial demands. As a result, there is a major impact on both the dairy industry and the environment including global warming. Recurrent mastitis is often attributed to the development of bacterial biofilms, which promote survival of sessile cells in hostile environments, and resistance to the immune system defense and antimicrobial therapy. Recently, we described the *in vitro* biofilm development on abiotic surfaces by bovine SDSD. In that work we integrated microbiology, imaging, and computational methods to evaluate the biofilm production capability of SDSD isolates on abiotic surfaces. Additionally, we reported that bovine SDSD can adhere and internalize human cells, including human epidermal keratinocyte (HEK) cells. We showed that the adherence and internalization rates of bovine SDSD isolates in HEK cells are higher than those of a SDSD DB49998-05 isolated from humans. *In vivo*, bovine SDSD can cause invasive infections leading to zebrafish morbidity and mortality. In the present work, we investigated for the first time the capability of bovine SDSD to develop biofilm *in vivo* using a murine animal model and ex-vivo on human HEK cells. Bovine SDSD isolates were selected based on their ability to form weak, moderate, or strong biofilms on glass surfaces. Our results showed that SDSD isolates displayed an increased ability to form biofilms on the surface of catheters implanted in mice when compared to *in vitro* biofilm formation on abiotic surface. A greater ability to form biofilm *in vitro* after animal passage was observed for the VSD45 isolate, but not for the other isolates tested. Besides that, *in vitro* scanning electron microscopy demonstrated that SDSD biofilm development was visible after 4 hours of SDSD adhesion to HEK cells. Cell viability tests showed an important reduction in the number of HEK cells after the formation of SDSD biofilms. In this study, the expression of genes encoding BrpA-like (biofilm regulatory protein), FbpA (fibronectin-binding protein A), HtrA (serine protease), and SagA (streptolysin S precursor) was higher for biofilm grown *in vivo* than *in vitro*, suggesting a potential role for these virulence determinants in the biofilm-development, host colonization, and SDSD infections. Taken together, these results demonstrate that SDSD can develop biofilms *in vivo* and on the surface of HEK cells causing important cellular damages. As SDSD infections are considered zoonotic diseases, our data contribute to a better understanding of the role of biofilm accumulation during SDSD colonization and pathogenesis not only in bovine mastitis, but they also shed some lights on the mechanisms of prosthesis-associated infection and cellulitis caused by SDSD in humans, as well.

## Introduction

*Streptococcus dysgalactiae* subsp. *dysgalactiae* (SDSD) has been considered an important bovine mastitis pathogen (Abdelsalam et al., 2010; Zadoks et al., 2011; Cervinkova et al., 2013; Rato et al., 2013) that causes severe economic repercussions over milk production. In addition, the association of SDSD with human infections has been reported (Koh et al., 2009; Park et al., 2012; Jordal et al., 2015; Chennapragada et al., 2018). Nevertheless, the role and importance of SDSD in human pathogenesis remain mostly unclear. Despite that, it is remarkable that SDSD is amongst the bacterial agents able to cause prosthetic joint infections (Park et al., 2012).

It is well known that biofilm is an important mechanism on the pathogenesis of medical device-associated infections, such as orthopedic prostheses (Ronin et al., 2022). Biofilms play an essential role in bacterial pathogenesis, promoting persistent infections and contributing to therapy failure. Biofilm formation involves various phases, including adhesion of the bacterial cells to the biotic and abiotic surfaces, in which diverse bacterial factors are involved (Kumar et al., 2017; Jamal et al., 2018). The great majority of the studies on bacterial biofilms have been based on *in vitro* growth on abiotic surfaces, which might be relevant for pathogens that grow on pacemakers, catheters, protheses and other implantable medical devices, increasing the risk of infections in hospital environments. Despite that, most bacterial host infections require biofilm formation on biotic surfaces as the initial stage of colonization or infection (Chao et al., 2017).

Additionally, host microenvironments, especially plasma proteins, are important for bacterial adherence to biotic or abiotic surfaces and biofilm formation during the process of a natural infection (Speziale et al., 2014). Therefore, *in vivo* models are important to gain a better understanding of the mechanisms involved in the development of biofilms and associated diseases. In the *in vivo* models of device-related infections (also called murine foreign body model), the foreign body can be inserted into the organ or into the subcutaneous space. The latter involves inserting a 1 cm segment of a catheter containing bacterium inoculum under animals’ skin (Prabhakara et al., 2011; Trøstrup et al., 2013; Kissoyan et al., 2016; See et al., 2016). Previous studies have shown that the murine model of prosthetic implant infection mediated by *Staphylococcus aureus* stimulates host responses like those observed in human infections (Prabhakara et al., 2011). Additionally, animal models have proved to be very useful providing excellent results in studies aimed at the development of new antibacterial agents and alternative therapies (Proetzel and Wiles, 2010; Cusumano et al., 2014). Other *in vivo* models of biofilm development have been described, such as those involving central venous catheter implantation in rats (Chauhan et al., 2016). This last model is more suitable for studies on the pathogenesis of bloodstream infections related to biofilm formation on catheter surfaces, while the murine model involving insertion of catheters into subcutaneous space would be more useful for studies on the role played by biofilm in foreign body infections (Prabhakara et al., 2011; Chauhan et al., 2016).

The ability of SDSD to form biofilms on abiotic surfaces was recently reported by us using confocal laser scanning microscopy, transmission electron microscopy and scanning electron microscopy (Alves-Barroco et al., 2019). We have also demonstrated for the first time that SDSD isolated from bovines can adhere to and internalize into human cells, including human epidermal keratinocyte (HEK) cells. Notably, the adherence and internalization rates of these SDSD isolates in HEK were higher than those of *S. pyogenes* and SDSD DB49998-05 (GCS-Si) isolates from humans (Roma-Rodrigues et al., 2016). ​Besides that, there are histological evidence that SDSD can cause invasive infections in a zebrafish model leading to morbidity and mortality (Alves-Barroco et al., 2018).

The first report of SDSD biofilm involvement in prosthetic joint infections in humans was provided in 2012. The patient was treated by re-implantation with an application of antibiotic-impregnated cement spacer (Park et al., 2012).

In the present work, we used BALB/c mice for a biofilm model *via* catheter implant to investigate the ability of SDSD isolates to form biofilms *in vivo*. Additionally, we compared *in vivo* and *in vitro* biofilm developments by SDSD isolates collected from bovines. We also investigated the capability of these isolates to develop biofilm on human keratinocyte (HEK) cells, since this bacterium can zoonotically infect humans causing, for example, cellulitis (Chennapragada et al., 2018). Finally, the expression profile of genes associated with virulence, including biofilm development and modulation, in other streptococci was analyzed both *in vivo* and *in vitro* to gain some insights on biofilm formation by SDSD isolates.

## Materials and methods

### Ethics

The animal experimentation was approved by the ethics committee on the use of animals from Centro de Ciências da Saúde, Universidade Federal do Rio de Janeiro, Brazil (#01200.001568/2013-87- CEAU).

### Biofilm formation assay on abiotic surfaces

The ability to form biofilms by 37 SDSD isolates from bovine clinical mastitis obtained between 2011 and 2013 [collection II, (Alves-Barroco et al., 2021)] was evaluated on polystyrene and glass surface (borosilicate test tubes) according to previously described protocols (Genteluci et al., 2015; Alves-Barroco et al., 2019; Xu et al., 2022). For a comparative analysis, 18 SDSD isolates from bovine clinical mastitis (collection I) (Rato et al., 2013; Alves-Barroco et al., 2019), obtained between 2002 and 2003, were included in the study. Sample collection design followed the international (Directive 2010/63/EU of the European parliament, on the protection of animals used for scientific purposes) and national (Decreto-Lei n° 113/2013) welfare regulations and guidelines (ARRIVE) was previously approved by the Portuguese “Direção Geral de Alimentação e Veterinária (DGAV)” (authorization document 0421/000/000/2013). In addition, the two authors have a level C FELASA certification (Federation of European Laboratory Animal Science Associations).

To evaluate the biofilm production on glass surfaces, the bovine SDSD isolates were streaked on blood agar plates and incubated at 37 °C for 18 h in a 5% (v/v) CO_2_ incubator. About 5 colonies were transferred to Tryptic Soy Broth (TSB; Becton, Dickinson and Company, Le Pont de Claix, France) supplemented with 0.5% (w/v) glucose and incubated at 37 °C until the middle of the exponential growth phase. The pure culture (OD_570_ = 0.6) was diluted 1:40 in a glass test tube (16 x 1,05 x 100 mm, NORMAX, Portugal) containing TSB supplemented with glucose (final volume 4 mL) and incubated at 37 °C for 20 h. Then, the supernatant was removed, and the glass tube washed with sterile saline solution [0.85% (w/v) NaCl]. The tubes were incubated at 65 °C for 1 h for drying. Biofilms were resuspended in 4 mL of saline and the OD at 600 nm measured. The isolates were defined as non-biofilm producers: OD_600_ ≤ 0.099, weak: OD_600_ between 0.1–0.299, moderate: between 0.3–0.599, or strong biofilm producers: OD_600_>0.600.

On polystyrene surfaces, after growing to the middle of the exponential phase, the bacterial culture (OD_570_ = 0.6) was diluted 1:2 in TSB supplemented with glucose in a 96 well plate (final volume 200 µL/well). The 96 well plate was sealed and incubated at 37 °C for 20 h. The supernatant was removed, and the wells washed with saline to remove non-adherent bacteria. Then, the plates were incubated at 65 °C for 1 h for drying and fixing biofilm. The biofilm was stained with crystal violet 1% (w/v) for 1 min. The wells were washed with sterile distilled water until the dye from the negative control-wells was completely removed. The OD_570_ of the stained biofilm was directly measured in a plate reader (Infinite M200, Tecan, Männedorf, Switzerland). Interpretation of biofilm formation was performed according to the criteria previously described (Alves-Barroco et al., 2019) and the isolates were therefore categorized as follows: non producer: OD ≤ OD_ctrl_, (OD_ctrl_ = 0.060); weak producer: OD_ctrl_ < OD ≤ 2 × OD_ctrl_; moderate producer: 2 × OD_ctrl_ < OD ≤ 4 × OD_ctrl_. strong producer: OD > 4 × OD_ctrl_

### SDSD biofilm formation on human keratinocyte cells

This assay was based on previously described protocols (Roma-Rodrigues et al., 2015) with few modifications. Bacteria were grown at 37°C in Todd Hewitt Broth (THB; Oxoid; Basingstoke, UK) supplemented with 0.5% (w/v) yeast extract until the middle exponential growth phase. The infection was started by adding bacterial suspension (containing 10^6^ bacterial cells) in Dulbecco’s Modified Eagle’s Medium (DMEM; ThermoFisher Scientific; Waltham, MA, USA) supplemented with 10% (v/v) fetal bovine serum (ThermoFisher Scientific) to 10^4^ human epidermal keratinocyte (HEK) cells (ATCC-PCS-200-010, ATCC, Manassas, VA, USA). The infected culture was incubated at 37 °C, 5% (v/v) CO_2_, and 99% relative humidity. After 2 h and 4 h of incubation, HEK cells were washed with phosphate buffer saline (PBS, 137 mM NaCl, 2.7 mM KCl, 10 mM Na_2_HPO_4_, and 1.8 mM KH_2_PO_4_, pH 7.4) (Sigma-Aldrich, St. Louis, MO, USA), to remove non-adhered bacteria, and then fixed with 2% (v/v) glutaraldehyde (Sigma-Aldrich) in PBS for 2 h at room temperature. The HEK cells were washed with PBS (three times), post-fixed with 1.0% (w/v) osmium tetroxide (Sigma-Aldrich) at 4°C for 1 h, and then processed as previously described (García-Pérez et al., 2003). The infected HEK cells were visualized using a scanning electron microscope (JEOL JSM-5400).

Viability of HEK cells was determined using MTS [3-(4,5-dimethylthiazol-2-yl)-5-(3-carboxymethoxyphenyl)-2-(4-sulfophenyl)-2H-tetrazolium, inner salt] assay as previously described (Fernandes et al., 2017). HEK cells were seeded in 96-well plates (ThermoFisher Scientific) and grown at 37 °C, 5% (v/v) CO_2_, and 99% relative humidity for 24 h, before incubations in the same conditions for 2 h, 4 h, and 6 h with SDSD cells or bacterial growth supernatant. After the incubation period, the culture medium was removed and, after washing HEK cells with PBS, fresh medium containing 10% MTS reagent (ThermoFisher Scientific) was added to each well. The 96-well plate was incubated at 37°C, in the same atmosphere, for 60 min. The absorbance (Abs) was measured in a microplate reader at 490 nm (Infinite M200, Tecan, Männedorf, Switzerland). The following equation was applied: cell viability (%) = 100 x [mean Abs of SDSD cells (or mean Abs bacterial growth supernatant)/mean Abs of control group without SDSD cells or bacterial growth supernatant].

### *In vitro* biofilm formation on the surface of an intravenous catheter segment

Exponentially growing SDSD cells in TSB supplemented with 0.5% (w/v) glucose were harvested by centrifugation and diluted in the fresh broth. A volume of 10 μL [containing 10^4^ colony forming units (CFU)] was injected into the lumen of a 1 cm segment of the polyurethane catheter (C-953-J-UDLM; Cook Inc., Bloomington, IN, USA). Then, the catheter was placed into the well of a 24-well plate and incubated for 72 h. To count the SDSD cells adhered, the catheter was washed with PBS twice, to remove non-adherent bacteria, and placed in fresh broth. After sonication (15 min; 38.5–40.5 kHz, in ice), the CFU/mL was determined using Todd Hewitt Agar (THA). Biofilm was assessed by counting the SDSD cells adhered to the catheter.

### *In vivo* biofilm formation

The *in vivo* assays using a mouse foreign-body model were performed as described in Genteluci et al., 2015. Briefly, young adult BALB/c mice (age between 8 to 10 weeks), obtained from NCAL-UFRJ (https://ccs.ufrj.br/paginas/sobre-o-ccs/coordenacoes/cambe), were anesthetized, and a subcutaneous incision was created to introduce a 1 cm segment of a polyurethane intravenous catheter containing 10 μL of the bacterial suspension (10^6^ CFU). The catheter was implanted subcutaneously (at least 1.5 cm from the incision). after 72 hours of infection, the animals were euthanized, and the catheter segments removed. After that, the catheter segments were washed with 0.15 M NaCl to remove any planktonic bacteria and placed in a tube containing 1 mL of saline. After sonication (15 min, 38.5–40.5 kHz, in ice), CFU/mL was determined using THA. Biofilm was assessed by counting the SDSD cells adhered to the catheter.

To investigate whether animal passage can increase the ability of SDSD to accumulate biofilms *in vitro*, cells collected from the catheter implanted in the mice were inoculated in TSB containing 0.5% (w/v) glucose at 37 °C for 18 h. Then, aliquots (with and without animal passage) were obtained to assess biofilm formation on the glass surface following the protocol described above.

### Reverse transcription quantitative PCR

Expression levels of genes associated with biofilm formation in other streptococci were evaluated using sessile cells recovered from *in vivo* and *in vitro* biofilms. RNA was extracted using NucleoSpin RNAII kit (Macherey-Nagel, Dueren, Germany) according to the manufacturer’s instructions to comparatively quantify transcripts of genes encoding BrpA-like (biofilm regulatory protein), FbpA (fibronectin-binding protein A), HtrA (serine protease), and SagA (streptolysin S precursor). The cDNA was synthesized using the SuperScript first-strand synthesis system (Invitrogen) according to the manufacturer’s instructions. The RT-qPCR reaction mixture (20 μL) contained NZY qPCR Green Master Mix (NZYTech, Lisbon, Portugal), 1 μL cDNA, and 0.5 μM of the forward and reverse primers described in **Table 1**. PCR conditions included an initial denaturation for 10 min at 95°C, followed by 30 cycles of amplification consisting of denaturation for 15 s at 95°C, and annealing for 30 s at 58°C and extension for 45 s at 60 °C. The critical Ct was defined as the cycle in which fluorescence becomes detectable above the background fluorescence. The expression levels were normalized using the 16S rRNA gene as an internal standard. Each assay was performed with at least three independent RNA samples.

**Table 1:** Primer sequences used for RT-qPCR analysis in this study.

Primer name	Sequence (5’-3’)	PCR product size (bp)	Reference
*brpA*-like			
for^a^	TGAAGCTAAGTTGAATGCTGC	534	Alves-Barroco et al., 2019
rev	GAACCACCATCAGACAAGGT		
*fbpA*-like			
for	CGCACCATTTTACCAGGCTC	376	Alves-Barroco et al., 2018
rev	TCAAGTCACTCGCTTGCTGA		
*htrA*			
for	TGCGACGATGAGTAAGATGG	218	This study
rev	TGACACCAGAACCTTGAGCA		
*sagA*			
for	TGGAGGTGTTAGGACATGAGG	192	This study
rev	CTTGCCTTTTCCGACGTTAG		
16S RNA			
for	ACCAAGGCGACGATACATAG	61	Genteluci et al., 2016
rev	GTGTCTCAGTCCCAGTGTG		

a for; forward, rev; reverse.

b bp; base pair.

### Statistical analysis

GraphPad Prism version 7.0 was used for statistical analysis. All data were expressed as mean ± SEM from at least three independent (biological) experiments. The statistical significance was determined for each data set using the student’s *t*-test, and statistical significance was considered when *p* < 0.05.

For comparison purposes, biofilm developments between SDSD isolates recovered from catheter implanted in mice (*in vivo*) and *in vitro* assay were both measured by CFU. In the case of biofilm developments by SDSD on glass surfaces before and after animal passage, biofilm growth was measured by OD determination.

## Results and discussion

### Biofilm formation assay on abiotic surfaces

**Figure 1:**
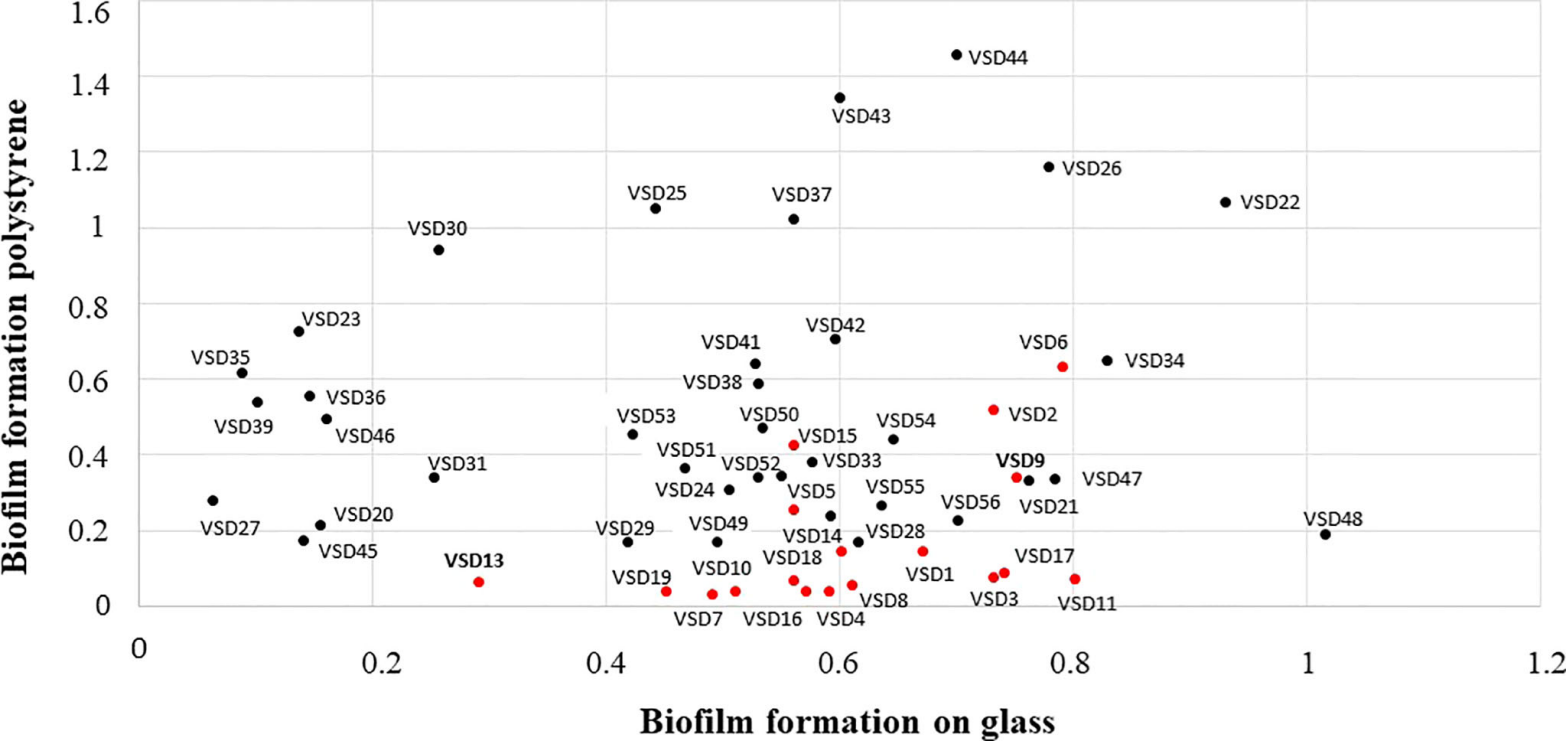
Comparison of the *in vitro* ability to form biofilms on abiotic surfaces by bovine SDSD isolates of clinical and subclinical mastitis in Portugal, during 2002-2003 (collection I, red circles) and 2011-2013 (collection II, black circles). Interpretation criteria for biofilm formation on polystyrene surface: i) non-producer: OD ≤ OD_ctrl_; ii) weak producer: OD ≤ OD_ctrl_ x 2; iii) moderate producer: OD_ctrl_ x 2 < OD ≤ OD_ctrl_ x 4; and iv) strong producer: OD > OD_ctrl_ x 4 Interpretation criteria for *in vitro* biofilm formation on glass surface: i) non-producer: OD_600_ ≤ 0.099; ii) weak producer; OD_600_ ≥ 0.1 ≤ 0.299; iii) moderate producer OD_600_: ≥ 0.3 ≤ 0.599; and iv) strong producer OD_600_ > 0.600. OD_ctrl_ = DO determined for the control.

As a first approach, the ability of 37 SDSD isolates (collection II) to form biofilms on glass and polystyrene surfaces was evaluated. For a comparative analysis, the results obtained for 18 SDSD isolates also obtained from bovines (collection I), shown in Alves-Barroco et al. (2019), were included in the study. Overall, despite some differences, the results obtained point to a high *in vitro* biofilm-forming ability by most SDSD isolates with isolates from the collection II showing a greater ability to accumulate biofilms than those from the collection I (**Figure 1**).

### SDSD biofilm formation on human keratinocyte cells

**Figure 2:**
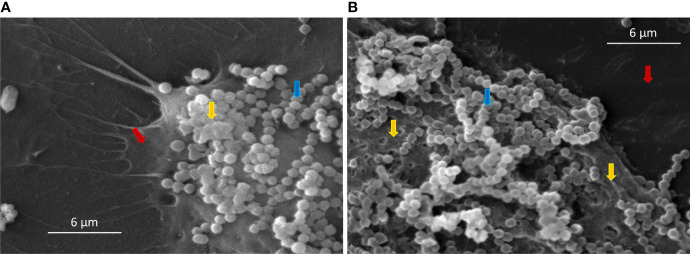
Scanning electron microscopy (SEM) of SDSD biofilms formed by VSD13 after **(A)** 2 h and **(B)** 4 h on human keratinocyte cells. Blue arrow: SDSD VSD13 cells; yellow arrow: formation of the extracellular polymeric matrix; red arrow: human keratinocyte cells.

HEK cells have an important role in host defense, providing a physical and immunological barrier against pathogenic bacteria. The adherence and/or invasion of Group G streptococci in HEK cells was associated with the severity of skin infections, e.g., necrotizing fasciitis (Siemens et al., 2015). We previously reported for the first time that bovine SDSD isolates are capable to adhere and internalize several human cells, including HEK cells (Roma-Rodrigues et al., 2016); Alves-Barroco et al., 2018. Here, the ability of SDSD isolates to form biofilms on HEK cells was analyzed by scanning electron microscope (**Figure 2**). Our results demonstrated the formation of an extracellular polymeric matrix by the growth of SDSD biofilms after 2 h of infection of HEK cells (**Figure 2A**). After 4 h of infection, it was possible to visualize a typical biofilm architecture (**Figure 2B**).

**Figure 3:**
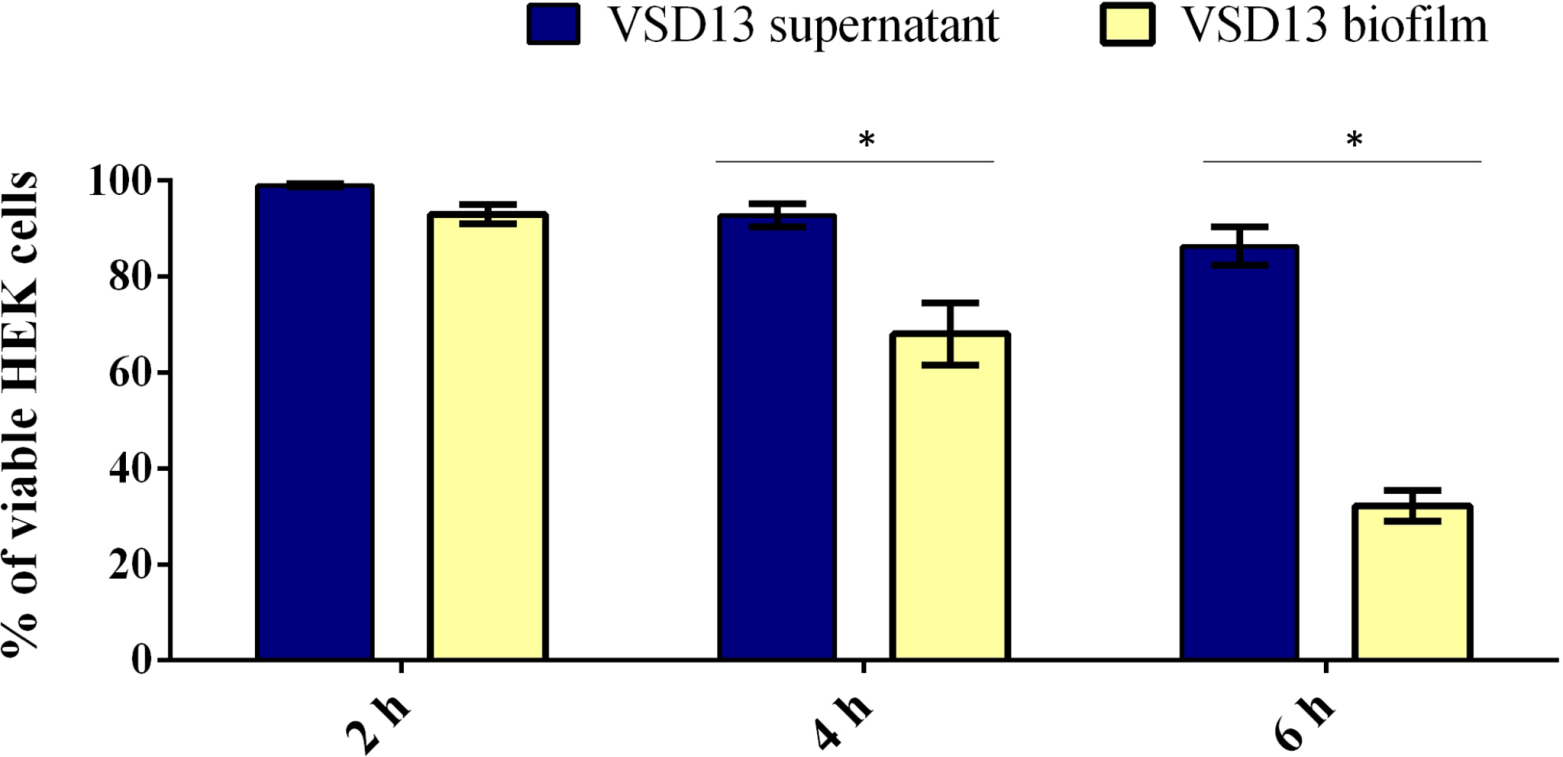
Viability of HEK cells exposure to SDSD VSD13 sessile cells or bacterial supernatants for 2 h, 4 h and 6 h. The following equation was applied: cell viability (%) = 100 x [mean Abs SDSD cells (or mean Abs bacterial growth supernatant)/mean Abs control group]. Data are the average of at least three independent (biological) assays with three technical replicates each. Error bars correspondent to standard deviation. Statistically significant differences were observed in the viability of HEK cells exposure to SDSD VSD13 sessile cells and bacterial supernatants at 4 h and 6 h, * *p* < 0.05.

Similar results were observed by Matsue and co-workers in investigations with other streptococci. They showed that after 2 h incubation of HEK cells with *Streptococcus dysgalactiae* subsp. *equisimilis* (SDSE), the percentage of adhered bacterial was on average 70%. Furthermore, the adherence of SDSE on HEK cells was about 10 times higher than that on polystyrene surfaces (Matsue et al., 2020). Previous studies have also shown the formation of biofilms by *Streptococcus pyogenes* in HEK cells. Visually, *S. pyogenes* biofilms formed on HEK cells were similar to biofilms on abiotic surfaces; however, *S. pyogenes* biofilms on HEK cells were more resistant to antimicrobial therapy (Marks et al., 2014). Marks and co-workers demonstrated that during coculture, the *S. pyogenes* biofilm extended about 20–30 µm above the HEK cells; however, *S. pyogenes* biofilms did not induce HEK cell death, as the keratinocytes layer remained intact during the experiment (Marks et al., 2014). Contrary to what was observed for *S. pyogenes* (Marks et al., 2014), SDSD biofilm induced a decline in viability of the HEK cells over time (**Figure 3**). After 6 h incubation with VSD13 isolate grown in biofilm condition or with its filtered culture supernatant, the viability of HEK cells was 32% and 86%, respectively. Our results also showed that the development of biofilms on the HEK cell monolayers exhibited greater cytotoxicity than extracellular products from bacterial growth (**Figure 3**). Thus, these data could indicate that SDSD biofilm may develop during the process of skin/soft tissue infections, suggesting that it might be important in the pathogenesis of human SDSD cellulitis.

Together, they suggest an important role for SDSD biofilm formation on HEK cells, which may contribute to the development of deeper tissue infections and bacterial dissemination. In fact, in recent years, the association of SDSD with human infections such as cellulitis has been reported (Chennapragada et al., 2018; Nathan et al., 2021), and one case of cellulitis rapidly progressed to septic shock (Nathan et al., 2021). Indeed, we have recently reported for the first time the resistance to conventional antibiotics associated with biofilm formation by SDSD (Alves-Barroco et al., 2022), which may complicate the treatment of some infections associated with this subspecies.

### *In vivo* Biofilm Formation

**Figure 4:**
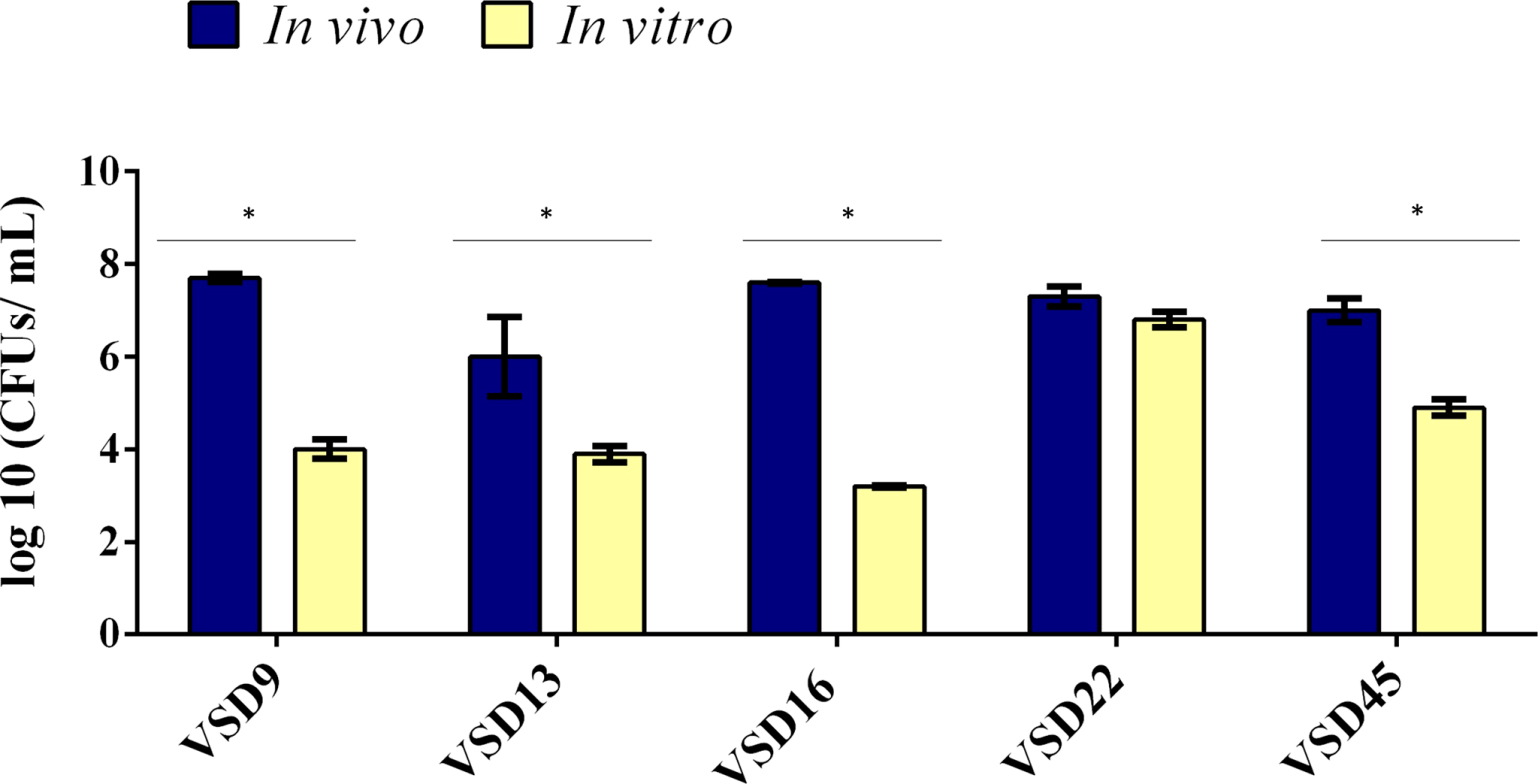
Comparison of biofilm development by bovine SDSD isolates recovered from catheter implanted in mice and by the *in vitro* assay. Statistically significant differences were observed in the formation of biofilms *in vivo* and *in vitro*, * *p* < 0.05.

To reduce the number of sacrificed animals and to compare *in vivo* and *in vitro* biofilm formation and accumulation, SDSD isolates were selected based on their *in vitro* ability to form strong (n=2; isolates VSD9 and VSD22; collection I and II, respectively), moderate (n=1; isolate VSD16), or weak (n=2; isolates VSD13 and VSD45; collection I and II, respectively) biofilms on the glass surface. All SDSD isolates tested, including weak biofilm producers *in vitro*, were able to develop biofilm *in vivo* (**Figure 4**).

The results showed an important increased ability to develop biofilm on catheter implanted in mice compared with the respective biofilm formed *in vitro*, except for the SDSD isolate VSD22, which already produced a very strong biofilm *in vitro*. Overall, the results suggest that the capability of SDSD isolated from bovines to develop strong biofilm *in vivo* is independent of the ability to form biofilms *in vitro* on abiotic surfaces. A possible limitation of this study is the fact that we used a collection of SDSD isolates from 2002 to 2013. However, it is important to emphasize that our results were not influenced by SDSD collection period, being similar for the isolates obtained in 2002-2003 or 2011-2013. Indeed, our data contribute for a better understanding of the pathogenic mechanisms of diseases not only in animals but also in humans, such as cellulitis and prosthetic joint infections that happened during these periods (Koh et al., 2009; Park et al., 2012).

**Figure 5:**
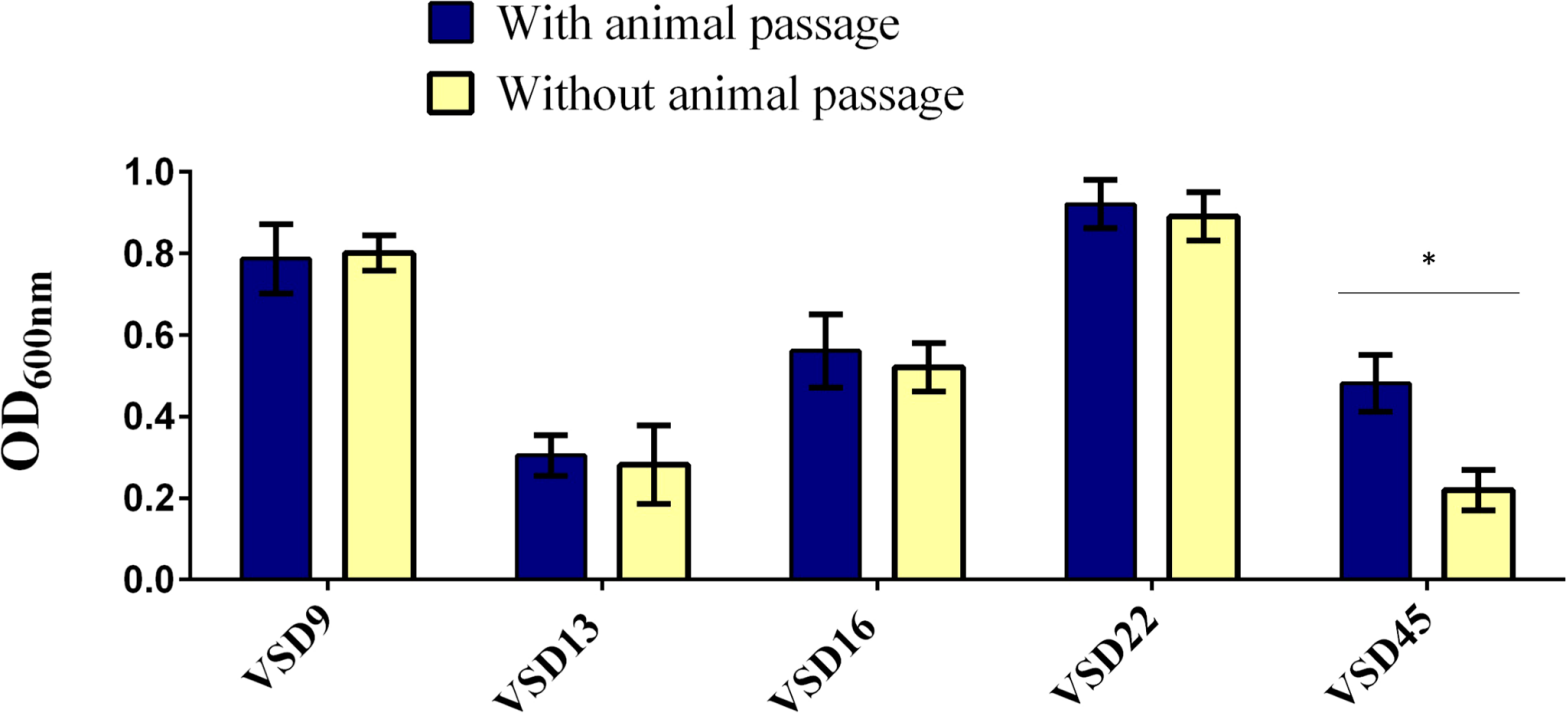
Biofilm development on glass surfaces by the representative biofilm producers with and without animal passage. Statistically significant differences were observed in the formation of biofilms after animal passage for VSD45, * *p* < 0.05. No significant differences were observed in the biofilm development on glass surfaces after animal passage for VSD9, VSD13, VSD16 and VSD22.

The quantification of *in vitro* versus *in vivo* biofilms (CFU/mL) varied, respectively, from 9.8 x10^3^ to 4.0 x10^7^ for VSD9 isolate, from 8.0 x10^3^ to 7.8 x10^6^ for VSD13 isolate, from 1.8 x10^3^ to 3.4 x10^6^ for VSD16 isolate, and from 7.4 x10^4^ to 6.4 x10^6^ for VSD45 isolate. An increased ability (approx. 2.2 times) to form biofilm *in vitro* (glass surface) after animal passage was observed for the SDSD isolate VSD45 (weak biofilm producer *in vitro*, **Figure 5**), but not for the other isolates tested.

Once the role of biofilms in infections was recognized, different *in vitro* and *in vivo* models of infections were developed. The *in vitro* models, although more simplistic, contributed to the current knowledge of the biofilm. These models are also used to investigate the role of genes involved in biofilm formation and to screen antimicrobial agents capable of disintegrating bacterial biofilms. However, *in vitro* models ignore important parameters and host factors. The *in vivo* models have contributed to a better understanding of bacterial adhesion, invasion and cytotoxicity factors, as well as the mechanisms involved in host inflammatory responses. There is no gold-standard model since each model can provide a specific answer. Information about models of biofilm-related infections and their applications is reviewed in Lebeaux et al., 2013.

The foreign-body mouse model used in the present work has also been successfully applied in previous studies with different bacterial species to analyze the ability of bacteria to form biofilms (Ferreira et al., 2012; Marks et al., 2014; Genteluci et al., 2015). Genteluci and co-workers showed that SDSE isolates can form biofilms *in vivo*, regardless of their ability to form biofilms *in vitro*. Marks and co-workers showed that *S. pyogenes* non-biofilm producers on abiotic surfaces ​can form biofilms on epidermal cells with characteristics similar to an *in vivo* colonization or infection (Marks et al., 2014). Although far from a complete understanding of the multiple factors that control the interactions between the pathogen and the host, animal models provide a better understanding of the biofilm within the context of the host. Taking all our data together, it can be concluded that *in vivo* growth increases the SDSD ability to form biofilms, possibly reflecting an important impact during some SDSD infections in bovine and in human hosts.

### Expression Profiles of Genes Associated With Biofilm Formation

**Figure 6:**
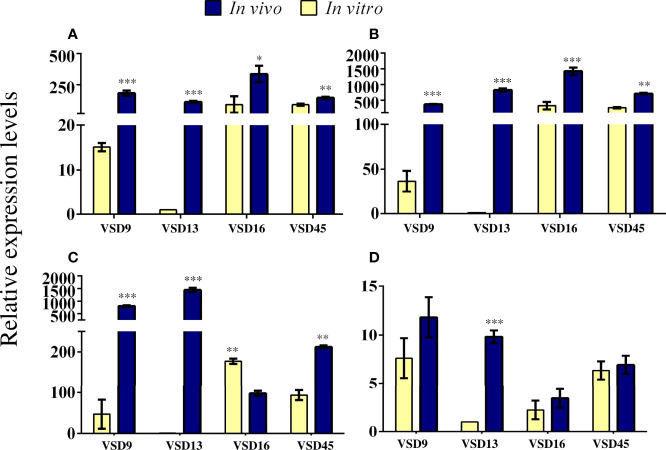
Relative expression levels of **(A)** *brpA*-like **(B)** *fbpA* **(C)** *htrA* and **(D)** *sagA* genes in sessile cells generated *in vivo* compared with those formed *in vitro*. RT-qPCR was expressed as the mean of three biologically independent experiments. The bar represents the standard deviation. The calibration sample was the cDNA for VSD13 biofilm grown *in vitro*. Statistically significant differences were observed for gene expression in SDSD biofilm grown *in vivo* or *in vitro*: * *p* < 0.05; ** *p* < 0.01; *** *p* < 0.001.

*In vivo* colonization by the group of pyogenic streptococci requires a series of interactions between the pathogen and the host, involving differential gene expression in both (Alves-Barroco et al., 2020). Our data revealed that *in vivo*, a similar number of sessile cells were recovered from SDSD isolates previously classified as weak and strong biofilm producers *in vitro*. This difference might be explained by changes in gene expression profiles associated with the regulation of biofilm formation (Marks et al., 2014). To test this hypothesis, we compared the expression of some biofilm-associated genes in sessile cells of SDSD isolates grown *in vivo* and *in vitro*; except for VSD22 isolate, as no difference was observed between *in vivo* and *in vitro* biofilm formation. Indeed, a remarkably increased expression was observed for the genes encoding BrpA-like (biofilm regulatory protein) and FbpA (fibronectin-binding protein A) for all bacterial biofilms collected from catheters recovered from the mice model (**Figures 6A**, **B**). The *brpA*-like gene was upregulated ~182, 112, 335, and 144-fold for VSD9, VSD13, VAS16, and VSD45, respectively, while *fbpA* was upregulated ~369, 822, 1419, and 708-fold for VSD9, VSD13, VAS16, and VSD45, respectively. The mRNA expression of *htrA* (encoding serine protease) was more dramatically increased for VSD9 (796-fold) and VSD13 (1441-fold) isolates, and mRNA expression of *sagA* (encoding streptolysin S percussor) for VSD13 isolate (~9.8-fold) (**Figures 6C**, **D**).

The role of the biofilm regulatory protein A (BrpA) in autolysis and cell division of the *Streptococcus mutans* has been shown. *In vitro*, the BrpA-deficient mutant of *S. mutans* maintained its adherence property, but the ability to form biofilms was considerably affected. Additionally, the deficiency of BrpA impaired cell envelope stress responses and acid and oxidative stress tolerance. (Bitoun et al., 2012; Bitoun et al., 2014). The biofilm-producing SDSD isolates carry a *brpA*-like gene, which expression level was associated with the ability to form biofilms *jn vitro* (Alves-Barroco et al., 2019). Therefore, our data showing a parallel increase of biofilm accumulation and *brpA*-like gene expression in the *in vivo* model corroborate a role played by this gene in the development of biofilm by SDSD.

Studies estimated that initial adherence to host cells is mainly mediated by adhesins, such as fibronectin-binding proteins, that allow adhesion to provide biofilm development on host tissues and/or bacterial internalization into host cells (Cue et al., 2000; Šmitran et al., 2018). Several fibronectin-binding proteins are produced by *S. dysgalactiae*, with different binding affinities and properties (Alves-Barroco et al., 2020). These proteins provide adherence to human cells such as fibroblasts and keratinocytes cells, contributing to biofilm development *in vivo* and consequently to persistent infections (Collin and Olsén, 2003; Brandt and Spellerberg, 2009). In this work, we observed an increased expression of fibronectin-binding protein A (*fbpA*) gene for sessile cells of SDSD grown *in vivo* compared with *in vitro* (**Figure 6B**). These results corroborate studies showing the important role of fibronectin-binding proteins in biofilm formation (Brandt and Spellerberg, 2009; O'Neill et al., 2009).

The high-temperature requirement protein A (HtrA, also known as DegP), is a serine protease widely distributed among streptococci (Alves-Barroco et al., 2020). HtrA homologs are responsible for the degradation of abnormal proteins in response to environmental stress. These proteins have also been identified in Gram-positive isolates (Kim and Kim, 2005). In *Streptococcus mutans*, the deletion of the *htrA* gene causes decreased ability to respond to environmental stress (Diaz-Torres and Russell, 2001). In *S. pyogenes*, the deletion of *htrA* gene affected the expression of several virulence genes (Lyon and Caparon, 2004). In addition to the proteolytic properties, this enzyme can adhere to the extracellular matrix of host tissues (Lyon and Caparon, 2004; Kim and Kim, 2005). Herein, differential expression of the *htrA* gene was observed between *in vitro* and *in vivo* biofilms. Isolates VSD9, VSD13 and VSD45 exhibited an increased *htrA* gene expression *in vivo* (**Figure 6C**). Interestingly, the VSD16 isolate exhibited a decreased expression of this gene (**Figure 6C**). Unlike other VSD isolates, such as VSD9 and VSD10, which produce extracellular matrices mostly composed of protein, VSD16 biofilm shows mucus-like material in the biofilm extracellular matrix (Alves-Barroco et al., 2019), suggesting that the matrix might be formed mostly by extracellular DNA (eDNA) or by complexes of eDNA and proteins (Alves-Barroco et al., 2019). Taken together, these results indicate that the VSD16 isolate may have a different biofilm formation pathway.

The *sagA* gene encodes the mature streptolysin S (SLS) toxin responsible mainly for the β-hemolytic activity among the pyogenic group of streptococci (Datta et al., 2005; Molloy et al., 2011). The SLS operon encodes the *sagA* gene (the structural propeptide), followed by genes that provide the conversion of SagA propeptide into SLS (*sagB* to *D*), leader cleavage (*sagE*), and transport across the membrane (*sagF* to *I*). The *S. pyogenes* SLS causes host soft-tissue damages, impacts phagocytes, and contributes to translocation across the epithelial barrier (Molloy et al., 2011). SLS also promotes programmed cell death and enhances inflammation in HEK cells (Flaherty et al., 2015). Studies revealed that SLS promotes host-associated biofilm formation (Vajjala et al., 2019), besides inducing mitochondrial damage and consequently macrophage death (Tsao et al., 2019). Datta and co-works reported that all SLS operon is required for the functional expression of streptolysin S (Datta et al., 2005). The loss of *Sag*B-I observed in SDSD isolates from bovine origin is associated with loss of β-hemolytic activity (Alves-Barroco et al., 2021); however, the *sagA* gene has been maintained in the bovine SDSD genome, which possibly indicates an additional function to the product of this gene (Alves-Barroco et al., 2021). Some studies suggested that SagA plays an important role in the regulation of several virulence determinants, including M proteins (Datta et al., 2005; Molloy et al., 2011). The mechanisms by which *sagA* mRNA regulates virulence in *Streptococcus* have been the subject of investigations. In the present study, a high and significant increase in *sagA* expression was observed *in vivo* for the sessile cells of VSD13 isolate (**Figure 6D**), suggesting that the regulation of *sagA* expression may differ in a strain-specific manner.

The multiple factors that control the interactions between the pathogen and the host is still far from being fully understood. Nonetheless, the animal models provide better knowledge of biofilm within the host context. Importantly, the *in vivo* biofilm growth of SDSD isolates trigger distinct stress pathways that lead to the upregulation of the *brpA*-like, *fbpA, htrA*, and *sagA* genes that may play an important role in the host colonization and infection. Although previous studies suggest that SDSD may have different host preferences (Porcellato et al., 2021), together, the results presented in the present work suggest that SDSD isolates from bovine origin are able to infect other hosts, and may have a potential zoonotic capability.

## Conclusions

To the best of our knowledge, the present study demonstrates for the first time in literature the ability of SDSD collected from bovines to form biofilm *in vivo* and suggests that the mechanism underlying biofilm development appears to be multifactorial. Despite that, the increase of *fbpA* transcripts in all sessile cells grown *in vivo* suggest a possible role for fibronectin-binding protein A in biofilm formation/accumulation. Indeed, the number of *brpA*-like gene transcripts was also higher in sessile cells corroborating a role of biofilm regulatory protein A in biofilm modulation of SDSD. Thus, future studies with knockout mutants are important to define exactly the role of each of these genes in biofilm development and SDSD-associated infections. In this work, we demonstrated that the capability of bovine SDSD to develop strong biofilm *in vivo* is independent on the ability to form biofilms *in vitro* on the abiotic surface. Moreover, we also provide data that show that bovine SDSD can form biofilms *ex vivo* on the surface of HEK cells causing important cellular damages. Due to SDSD ability to cause severe zoonotic infections, our data contribute to a better understanding of the role of biofilm accumulation during SDSD colonization and pathogenesis in human skin infections and possibly in bovine mastitis as well. Additional studies are required toward a better understanding of the mechanisms associated with the regulation of biofilm formation by SDSD isolates and the precise role of biofilm development in SDSD infections.

## Data availability statement

The original contributions presented in the study are included in the article/supplementary material. Further inquiries can be directed to the corresponding authors.

## Ethics statement

The animal study was reviewed and approved by Centro de Ciências da Saúde, Universidade Federal do Rio de Janeiro, Brazil (#01200.001568/2013-87- CEAU).

## Author contributions

AMF and ARF were involved in the study conception and design, coordination, and revision of the final version of the manuscript. CA-B contributed to biofilm formation assay *in vivo* and *in vitro* and analysis of the expression of genes associated with the formation of biofilms, and statistical analysis. CA-B and APM contributed to analysis of SDSD biofilm formation in human keratinocyte cell. CA-B, MA, BF-C and SF contributed to subcutaneous catheter implantation in the animal model. CA-B and MG contributed to biofilm formation on glass and polystyrene assay. CA-B wrote the draft version of the manuscript. All authors read and corrected the manuscript.

## Funding

This work was supported in part by grants # 307672/2019-0 from Conselho Nacional de Desenvolvimento Científico e Tecnológico (CNPq); # E-26/200.952/2021, # E-26/010.002435/2019 and # E-26/010.001280 from Fundação de Amparo à Pesquisa do Rio de Janeiro (FAPERJ); and # 001 from Coordenação de Aperfeiçoamento de Pessoal de Nível Superior (CAPES). This work is also financed by national funds from FCT - Fundação para a Ciência e a Tecnologia, I.P., in the scope of the project UIDP/04378/2020 and UIDB/04378/2020 of the Research Unit on Applied Molecular Biosciences - UCIBIO and the project LA/P/0140/2020 of the Associate Laboratory Institute for Health and Bioeconomy - i4HBand also by projects PTDC/CVT-EPI/4651/2012 and PTDC/CVT-EPI/6685/2014. FCT-MEC is also acknowledged for grant SFRH/BD/118350/2016 to CA-B.

## Conflict of interest

The authors declare that the research was conducted in the absence of any commercial or financial relationships that could be construed as a potential conflict of interest.

## Publisher’s note

All claims expressed in this article are solely those of the authors and do not necessarily represent those of their affiliated organizations, or those of the publisher, the editors and the reviewers. Any product that may be evaluated in this article, or claim that may be made by its manufacturer, is not guaranteed or endorsed by the publisher.
